# Holliday junction recognition protein (HJURP) could reflect the clinical outcomes of lung adenocarcinoma patients, and impact the choice of precision therapy

**DOI:** 10.3389/fgene.2024.1475511

**Published:** 2024-11-22

**Authors:** Xixi Gao, Yingqing Zhang, Ming Zhang, Yuejiao Sun

**Affiliations:** Department of Respiratory, The Affiliated Hospital of Jiaxing University, Jiaxing, Zhejiang, China

**Keywords:** HJURP, lung adenocarcinoma, prognosis, immune infiltration, genetic alterations

## Abstract

**Background:**

Lung adenocarcinoma (LUAD) is the most prevalent subtype of non-small cell lung cancer (NSCLC), characterized by poor prognosis and a high mortality rate. Identifying reliable prognostic biomarkers and potential therapeutic targets is crucial for improving patient outcomes.

**Methods:**

We conducted a comprehensive analysis of HJURP expression in LUAD using data from four cohorts: TCGA-LUAD (n = 453), GSE31210 (n = 226), GSE68465 (n = 442), and GSE72094 (n = 386). Univariate Cox regression analysis was employed to identify prognostic genes, with Kaplan-Meier survival analysis used to assess the predictive power of HJURP. Functional enrichment analyses were performed using MetaScape and FGSEA, and spatial transcriptomics and single-cell sequencing data were analyzed to explore HJURP’s distribution and potential functions. Additionally, correlations between HJURP expression and genetic alterations, immune cell infiltration, and potential therapeutic responses were evaluated.

**Results:**

HJURP was identified as a significant prognostic biomarker in all four cohorts, with high expression associated with increased risk of overall survival (OS) death (TCGA-LUAD: HR = 1.93, 95% CI: 1.321–2.815, *P* < 0.001; GSE31210: HR = 2.75, 95% CI: 1.319–5.735, *P* = 0.007; GSE68465: HR = 1.57, 95% CI: 1.215–2.038, *P* < 0.001; GSE72094: HR = 2.2, 95% CI: 1.485–3.27, *P* < 0.001). Functional analyses indicated that HJURP is involved in DNA metabolic processes, cell cycle regulation, and mitotic processes, with significant activation of pathways related to MYC targets, G2M checkpoint, and DNA repair. High HJURP expression was associated with higher mutation frequencies in TP53, CSMD3, TTN, and MUC16, and positively correlated with pro-inflammatory immune cell infiltration and several immune checkpoints, including PD-L1 and PD-L2. Chemotherapeutic agents such as gefitinib and sorafenib were predicted to be effective against high HJURP-expressing tumors.

**Conclusion:**

HJURP is a pivotal biomarker for LUAD, consistently associated with poor prognosis and advanced disease stages. Its high expression correlates with specific genetic alterations and immune profiles, highlighting its potential as a therapeutic target. Future studies should validate these findings in larger cohorts.

## Introduction

According to the latest data released by the International Agency for Research on Cancer and the World Health Organization, there were 24.80 million new cancer cases and 18.17 million cancer-related deaths worldwide in 2022. Lung cancer had an incidence rate of 12.4%, making it the second most common cancer globally, and a mortality rate of 18.7%, the highest among all cancers (Bray et al., 2024). As for China, lung cancer also accounts for the first frequently tumor of both diagnosis and death, there are about 870,982 new cancer cases and 766,898 cancer-related deaths in 2022, both the incidence rate and mortality rate are significantly severe than global level (Xia et al., 2022). The two main types of lung cancer are small cell lung cancer (SCLC) and non-small cell lung cancer (NSCLC), with NSCLC accounting for approximately 85% of all lung cancer cases. Lung adenocarcinoma (LUAD) is the most prevalent subtype of NSCLC, accounting for approximately 40%–50% of all lung cancer cases (Herbst et al., 2018; Lahiri et al., 2023). Clinically, LUAD often presents with symptoms such as persistent cough, chest pain, and shortness of breath, and it is frequently diagnosed at an advanced stage due to its asymptomatic early course (Ruano-Ravina et al., 2020). Despite significant advancements in various treatments such as surgery, radiation therapy, chemotherapy, targeted therapy, and immunotherapy, the 5-year survival rate remains below 18% (Siegel et al., 2021).

The incidence of LUAD is influenced by factors such as smoking, genetic susceptibility, and environmental exposures (e.g., air pollution and occupational hazards) (Wu et al., 2022). Interestingly, LUAD is more common in non-smokers compared to other lung cancer subtypes, suggesting that genetic and environmental factors play significant roles in its development beyond smoking. A Japanese case-control study showed that smoking had a greater impact on squamous cell carcinoma (SqCC) and SCLC than on LUAD, with odds ratios (OR) of 9.08 and 10.18 for SqCC and SCLC, respectively, compared to an OR of 2.14 for AD in men (Seki et al., 2013). Air pollution, particularly particulate matter with an aerodynamic diameter ≤2.5 μm (PM2.5), is another significant risk factor for lung cancer. Long-term exposure to high concentrations of PM2.5 can induce chronic inflammatory responses and repeated particulate deposition, thereby disrupting the lung cells’ self-repair capabilities and increasing lung cancer risk (Lequy et al., 2021; Wang et al., 2022). A study from China found that lung adenocarcinoma is the most common genetic type, with its proportion rising, especially among non-smokers. The study also identified severe urban pollution and being female as additional risk factors for LUAD (Li et al., 2022). Another study indicated that for every 10 μg/m^3^ increase in PM2.5, the lung cancer mortality rate increases by 6.2% (Chung et al., 2021). Approximately 50%–70% of LUAD patients are found to have driver gene mutations, although the exact percentage may vary depending on the study or patient population (Saito et al., 2016). Mutations in genes such as TP53, EGFR, KRAS, BRAF, and ALK, as well as arm-level copy number alterations (CNA) and loss of heterozygosity in HLA, are increasingly frequent in the development of lung adenocarcinoma. These genomic alterations drive tumor growth and serve as targets for specific therapies (Kaneko et al., 2024). EGFR is a transmembrane signaling receptor that plays a central role in various cellular processes, including proliferation, migration, adhesion, and invasion. EGFR is overexpressed in several epithelial cancers, including NSCLC, making it a proposed therapeutic target. Initial clinical trials of oral EGFR tyrosine kinase inhibitors (TKIs), such as erlotinib and gefitinib, demonstrated moderate efficacy in unselected NSCLC patients’ post-chemotherapy, with response rates around 10% and a median survival extension of 2 months compared to placebo (Kim et al., 2008; Shepherd et al., 2005).

The tumor microenvironment (TME) predominantly comprises various subpopulations of T and B lymphocytes, dendritic cells (DCs), macrophages, neutrophils, and myeloid-derived suppressor cells (MDSCs) (Belli et al., 2018). The equilibrium between pro-tumorigenic and anti-tumorigenic factors within the TME dictates tumor progression. Numerous immune cells, such as M2 macrophages and regulatory T cells (Tregs), contribute to tumor immune evasion (Pitt et al., 2016). Recent days, a study utilizing the data of 361,929 cells analyzed with single-cell RNA sequencing that from 35 LUAD samples, reveling an immune cell module associated with tumor mutational burden (TMB), cancer-testis antigens, TP53 mutations, and an enhanced response to immune checkpoint inhibitors (ICIs) in patients with even median TMB. This module also correlated with the cancer-associated fibroblast (CAF) score and inversely correlated with the fibroblast score (Leader et al., 2021). Thus, a strong interconnection exists between tumor immune infiltration, tumor gene expression patterns, and the TME.

Therefore, elucidating the molecular mechanisms of LUAD development and progression, especially regarding the immune phenotypes that clarify tumor-immune interactions, and identifying new immunotherapy-related targets is of paramount importance. In the current study, we aimed to identify key genes that can reflect clinical outcomes by including multiple LUAD sequencing cohorts. We analyzed their potential roles from perspectives such as alterations in cell signaling pathways and gene mutations, in order to identify potential new targets for clinical treatment of LUAD.

## Methods

### Patient summary

A cohort of 453 patients from The Cancer Genome Atlas (TCGA)-LUAD was initially included for analysis. Each patient possessed comprehensive gene expression profiles along with corresponding clinical information. All data were obtained using the R package “TCGAbiolinks” (Colaprico et al., 2016). In addition, several cohorts released on Gene Expression Omnibus (GEO) database with overall survival (OS) time were also enrolled, including GSE31210, GSE68465, GSE72094, GSE11117, GSE11969, GSE13213, GSE = 42,127, GSE19188, GSE63459, GSE29016. GSE40791 and GSE31547 contains the gene expression matrix of both normal and tumor sample, and also be employed to compare the different expression. Importantly, patients with an overall survival (OS) time of less than 1 month were excluded to mitigate potential bias. For the TCGA-LUAD gene expression profile, genes with zero expression in more than 10% of samples were also excluded. The count data were converted to transcripts per kilobase million (TPM) values, followed by a log2(TPM+1) transformation for subsequent analysis (Lu et al., 2019). All the gene expression data ranges from 0 to 20 after scale. All the GEO cohorts can be downloaded from the Gene Expression Omnibus (https://www.ncbi.nlm.nih.gov/geo/).

### Identify the pivotal prognostic gene

Univariate Cox analysis was employed to identify prognostic genes in the TCGA-LUAD and three GEO cohorts, selecting those with a hazard ratio (HR) greater than 1.5 and a *p*-value less than 0.05. Subsequently, a Venn diagram was utilized to display the consensus prognostic genes for further analysis. Kaplan-Meier curves were generated to compare overall survival (OS) using the log-rank test. A meta-analysis based on the HR and 95% confidence intervals (95% CI) was calculated to uncover the overall prognostic value.

### Functional signaling enrichment analysis

We calculated the correlations among genes with selected gene by Pearson correlation test, and the signaling enrichment of the top 200 genes were performed by MetaScape (http://metascape.org/) (Zhou et al., 2019). We evaluated the activated pathways using fast gene set enrichment analysis (fgsea, https://github.com/ctlab/FGSEA/). Initially, GSEA was conducted by ranking the input molecular readouts, followed by calculating the pathway enrichment score through a running-sum statistic. This method increases the score if a feature falls into the target pathway and decreases it otherwise. The final score represents the maximum deviation from zero observed during the random walk, normalized by computing the z-score of the estimate compared to a null distribution derived from random permutations. CancerSEA (Yuan et al., 2019) (http://biocc.hrbmu.edu.cn/CancerSEA) is the first dedicated database designed to comprehensively resolve the distinct functional states of cancer cells at the single-cell level. It provides a cancer single-cell functional state atlas encompassing 14 functional states—stemness, invasion, metastasis, proliferation, EMT, angiogenesis, apoptosis, cell cycle, differentiation, DNA damage, DNA repair, hypoxia, inflammation, and quiescence—across 41,900 cancer single cells from 25 cancer types. We downloaded these gene sets and utilized the z-score algorithm in the R package GSVA to calculate the functional status of the 14 gene sets, with the values for each gene set expressed as z-scores. Pearson correlation analysis was then employed to determine the statistical correlation of genes with each gene-based z-score.

### Genetic alteration, immunocyte infiltration and precision therapy

We compared the different gene expression among wild type and mutated samples in TCGA-LUAD cohort by TIMER 2.0 (http://timer.cistrome.org/). The correlation between expression of selected gene and other mutated genes were also calculated by Pearson correlation analysis, and further validated in GSE26939 and GSE72094. Correlation between selected gene and immunocyte infiltration was also evaluated by TIMER 2.0, we also assessed the distribution of high and low selected gene expression TCGA-LUAD sample among six immune subtypes, including wound healing (C1), IFN-γ dominant (C2), inflammatory (C3), lymphocyte depleted (C4), immunologically quiet (C5), and TGF-β dominant (C6) (Thorsson et al., 2018). The potential response of immunotherapy and chemotherapy evaluated by BEST (https://rookieutopia.com/) (Zaoqu et al., 2023). IMvigor210 cohort contains 348 patients that received the therapy of PD-L1 blockade with atezolizumab in metastatic urothelial cancer (Mariathasan et al., 2018), the clinical information and gene expression data can be accessed from http://research-pub.gene.com/IMvigor210CoreBiologies/. The data of Wolf 2021 cohort can be assessed from GSE173839.

### Spatial transcriptomics and single-cell sequencing

To observe the protein level and localization of specific gene, we checked the Human Protein Atlas (HPA, https://www.proteinatlas.org/) website, of which provide the picture of immunofluorescent staining. To further explore the distribution of specific gene in LUAD, we also employed the data from spatial transcriptomics and single-cell sequencing. We acquired the data about samples from patients with brain metastasis of non-small cell lung carcinoma (GSE179572) (Sudmeier et al., 2022), to evaluate the spatial distribution of specific gene. To accurately assess the cellular composition of each spot on the 10x Visium slides, we employed deconvolution analysis. This method leverages spatial transcriptomics and single-cell transcriptomics data, with particular consideration given to the specific cancer type. Based on the preceding deconvolution results, we calculated the predominant cell type in each microregion and visualized the highest cellular content in each microregion using the SpatialDimPlot function from the Seurat package (Supplementary Table S1). The SpatialFeaturePlot function from the Seurat package was utilized to visualize the gene expression landscape in each microregion. Spearman correlation analysis was performed to calculate the correlations between cellular contents across all spots and between cellular content and gene expression. The results were visualized using the linkET package. The data of single-cell profiling of advanced non-small cell lung cancer (GSE148071) (Wu et al., 2021) was also download for the further analysis. The analysis and virilization of single-cell data based on TISCH2 (http://tisch.comp-genomics.org/) (Han et al., 2023). The cell type of malignant and alveolar was collected from the source data, while the marker genes of immunocytes provided in Supplementary Table S2.

### Statistical analyses

All the statistical analyses were performed by the R version 4.2.2. Student’s t-test was applied to compare two groups if the data is normally distributed for continuous data, otherwise Wilcoxon rank-sum test will be used. For the comparison of continuous data among more than two groups, Kruskal–Wallis test was utilized. As for categorical data, Chi-square test and Fisher’s exact test was conducted. Pearson correlation coefficient analysis was used to calculate the correlation of HJURP with other types of data. The receiver operating characteristic (ROC) area under the curve (AUC) was performed to access the stability of prediction. To find out the independent risk factors, univariate analysis and multivariate analysis were performed. *p* < 0.05 was considered statistically significant.

## Results

### HJURP shows the prognostic value for LUAD

**FIGURE 1:**
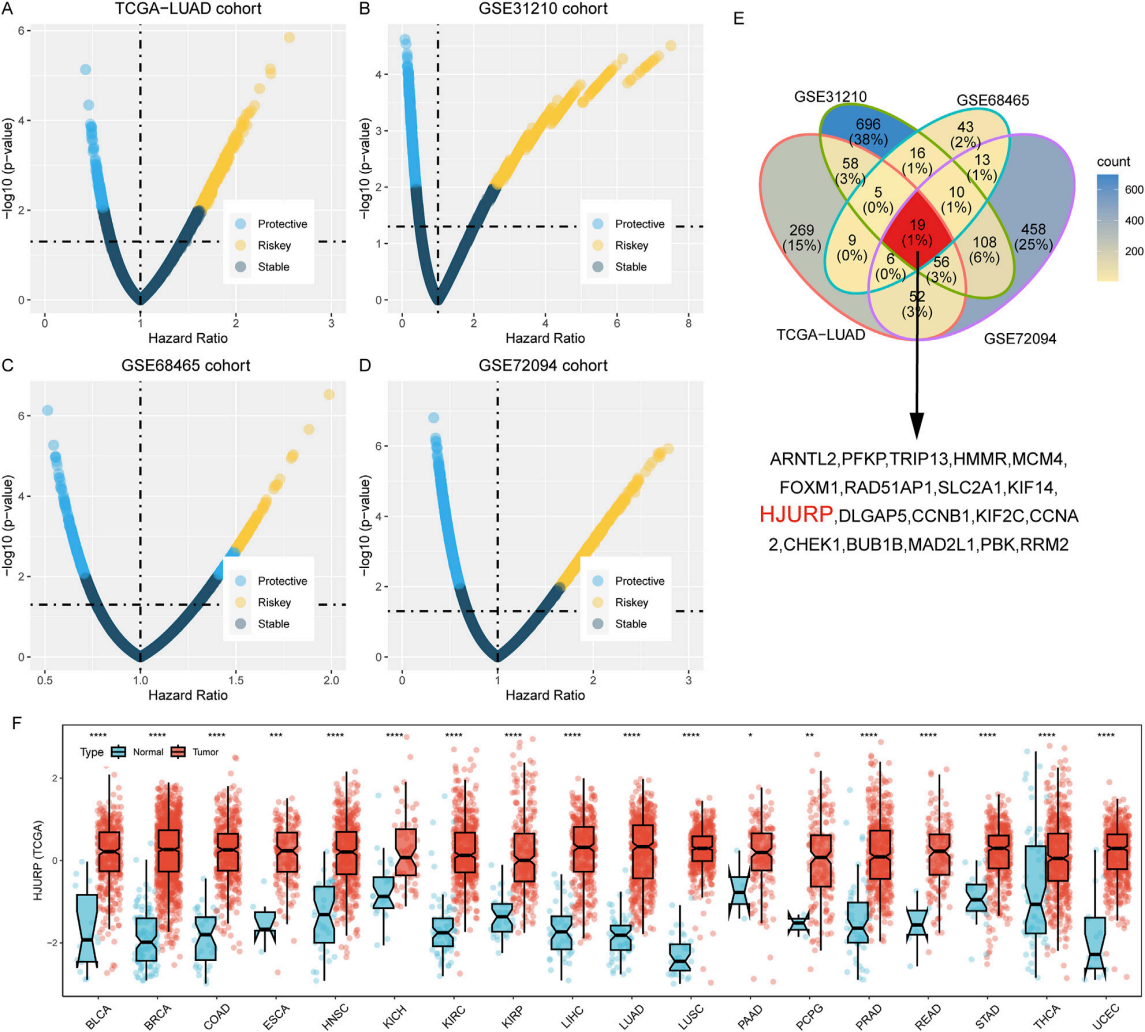
Identification of Prognostic Genes in Four Cohorts Using Univariate Cox Regression Analysis **(A–D)**. Univariate cox regression analysis across TCGA-LUAD **(A)**, GSE31210 **(B)**, GSE68465 **(C)**, and GSE72094 **(D)**; **(E)**. Merged analysis of the risk genes identified across the four cohorts by Venn plot; **(F)**. Pan-cancer analysis of HJURP expression among tumor and normal tissues across 18 types of tumors. BLCA: Bladder Urothelial Carcinoma; BRCA: Breast invasive carcinoma; COAD: Colon adenocarcinoma; ESCA: Esophageal carcinoma; HNSC: Head and Neck squamous cell carcinoma; KICH: Kidney Chromophobe; KIRC: Kidney renal clear cell carcinoma; KIRP: Kidney renal papillary cell carcinoma; LIHC: Liver hepatocellular carcinoma; LUAD: Lung adenocarcinoma; LUSC: Lung squamous cell carcinoma; PAAD: Pancreatic adenocarcinoma; PCPG: Pheochromocytoma and Paraganglioma; PRAD: Prostate adenocarcinoma; READ: Rectum adenocarcinoma; STAD: Stomach adenocarcinoma; THCA: Thyroid carcinoma; UCEC: Uterine Corpus Endometrial Carcinoma.

To identify prognostic genes, we employed univariate Cox regression analysis in four cohorts: TCGA-LUAD, GSE31210, GSE68465, and GSE72094. We assessed the prognostic predictive power of all genes, applying a predefined threshold (HR > 1.5, *p* < 0.01). This analysis revealed 474 risk genes in the LUAD cohort (Figure 1A), 968 risk genes in the GSE31210 cohort (Figure 1B), 121 risk genes in the GSE68465 cohort (Figure 1C), and 722 risk genes in the GSE72094 cohort (Figure 1D). Subsequently, we performed a merged analysis of the risk genes identified across the four cohorts by Venn plot (Figure 1E), uncovering 19 genes consistently associated with accelerated tumor progression. For all the 19 genes, HJURP shows the secondary rank with its expression fold change of 3.71 compared between tumor and normal samples, only less than KIF14, and for the expression level, HJURP have the higher level than KIF14 (Supplementary Figure S1). Holliday junction recognition protein (HJURP) is a key molecular chaperone for centromere protein A (CENP-A), which is essential for chromosome separation during mitosis and cell cycle regulation. Subsequently pan-cancer analysis, we also revealed that high HJURP expression in 18 types of tumors, including LUAD (all *p* < 0.05, Figure 1F).

Several studies based on clinical tumor samples also confirmed the risk of HJURP to the tumorigenesis or development of cholangiocarcinoma (Yang et al., 2022), colorectal cancer (Kang et al., 2020), pancreatic cancer (Wang et al., 2020), triple-negative breast cancer (Mao et al., 2022) and hepatocellular carcinoma (Chen et al., 2018). While HJURP has indeed been reported in other types of cancers, its role in LUAD remains underexplored, which presents a significant opportunity for novel discoveries. Our decision to focus on HJURP stems from its well-documented function in maintaining chromosomal stability through the centromeric loading of CENP-A, a key process that is often dysregulated in various cancers, including lung cancer.

**FIGURE 2:**
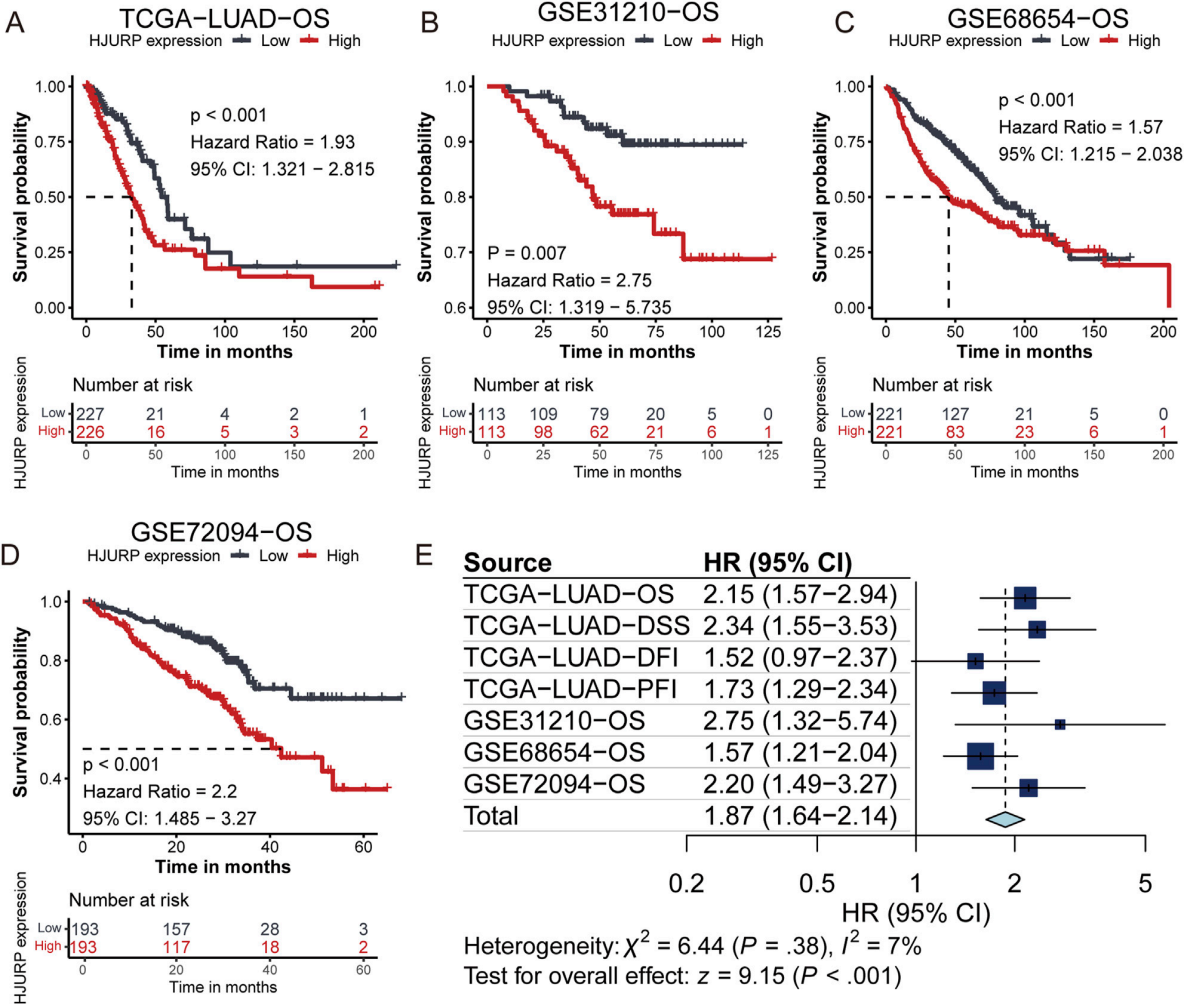
Kaplan-Meier Survival Analysis of HJURP Gene Expression Across Different Cohorts. **(A–D)**. Kaplan-Meier showing the prognostic value of HJURP across TCGA-LUAD **(A)**, GSE31210 **(B)**, GSE68465 **(C)**, and GSE72094 **(D)**; **(E)**. Meta-analysis of HJURP gene expression impact on clinical outcomes.

We utilized Kaplan-Meier survival analysis to illustrate the predictive power of the HJURP gene across different cohorts. In the TCGA-LUAD cohort, patients with high HJURP expression had a 1.93-fold higher risk of overall survival (OS) death compared to those with low HJURP expression (95% CI: 1.321–2.815, *p* < 0.001, Figure 2A). In the GSE31210 cohort, the high HJURP expression group had a 2.75-fold higher risk of OS (95% CI: 1.319–5.735, *p* = 0.007, Figure 2B). In the GSE68654 cohort, high HJURP expression was associated with a 1.57-fold higher risk of OS (95% CI: 1.215–2.038, *p* < 0.001, Figure 2C). In the GSE72094 cohort, high HJURP expression corresponded to a 2.2-fold higher risk of OS (95% CI: 1.485–3.27, *p* < 0.001, Figure 2D).

Furthermore, we analyzed the impact of HJURP gene expression on various clinical outcomes within the TCGA-LUAD cohort. High HJURP expression was indicative of poorer disease-specific survival (DSS), disease-free interval (DFI), and progression-free interval (PFI). Through a meta-analysis, we integrated the prognostic predictive power of HJURP across different cohorts and found that patients with high HJURP expression had a 1.87-fold higher risk of adverse prognostic outcomes compared to those with low expression (Figure 2E). These findings collectively demonstrate that high HJURP expression is significantly associated with decreased survival rates in multiple cohorts.

### HJURP expression is closely related to various clinical phenotypes

**FIGURE 3:**
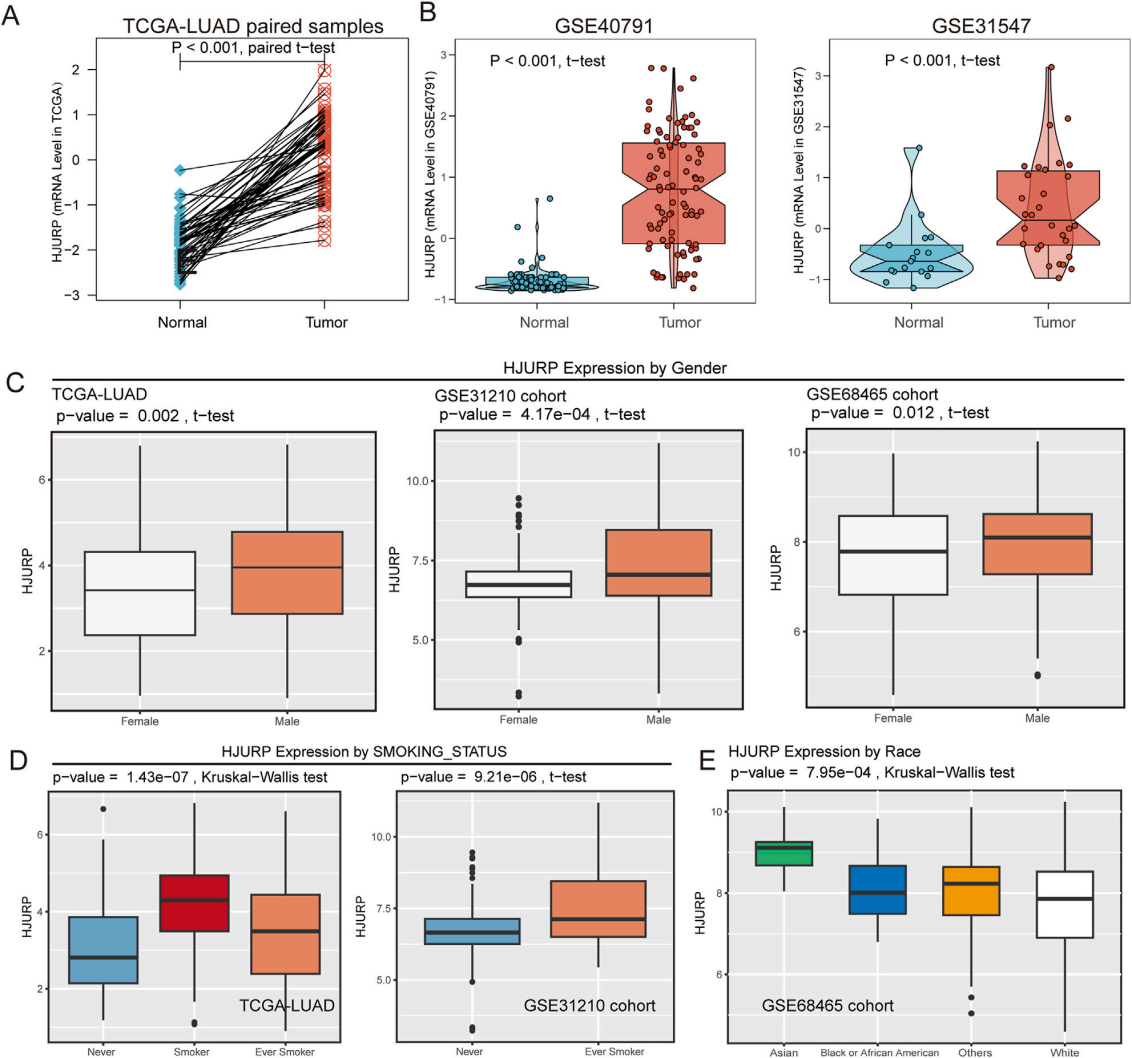
HJURP Expression Analysis Across Pan-Cancer, TCGA, and Validation Cohorts. **(A)** HJURP expression in TCGA-LUAD tumors and paired adjacent normal samples; **(B)** Validation of HJURP expression in GSE40791 and GSE31547 cohorts; **(C)** HJURP expression comparing by gender subgroup in TCGA-LUAD, GSE31210, and GSE68465 cohorts; **(D)** HJURP expression comparing by smoking status in TCGA-LUAD and GSE31210 cohorts; **(E)** HJURP expression comparing by race in the GSE68465 cohort.

In the TCGA cohort, HJURP expression was significantly elevated in tumors, as compared between paired tumor and adjacent normal samples (*p* < 0.001, Figure 3A). This finding was further validated in the GSE40791 and GSE131547 cohorts (*p* < 0.001, Figure 3B). In clinical phenotype-related analyses, we found that HJURP expression was significantly higher in male patients compared to female patients (TCGA-LUAD: *p* = 0.002, GSE31210: *p* < 0.001, GSE68654: *p* = 0.012, Figure 3C). Moreover, HJURP expression was closely related to smoking status, with higher expression observed in current and ever smokers with lung cancer (TCGA-LUAD: *p* < 0.001, GSE31210: *p* < 0.001, Figure 3D), suggesting that smoking may influence tumorigenesis by upregulating HJURP expression. Additionally, we found that Asians had higher HJURP expression compared to other ethnicities (GSE68654: *p* < 0.001, Figure 3E).

**FIGURE 4:**
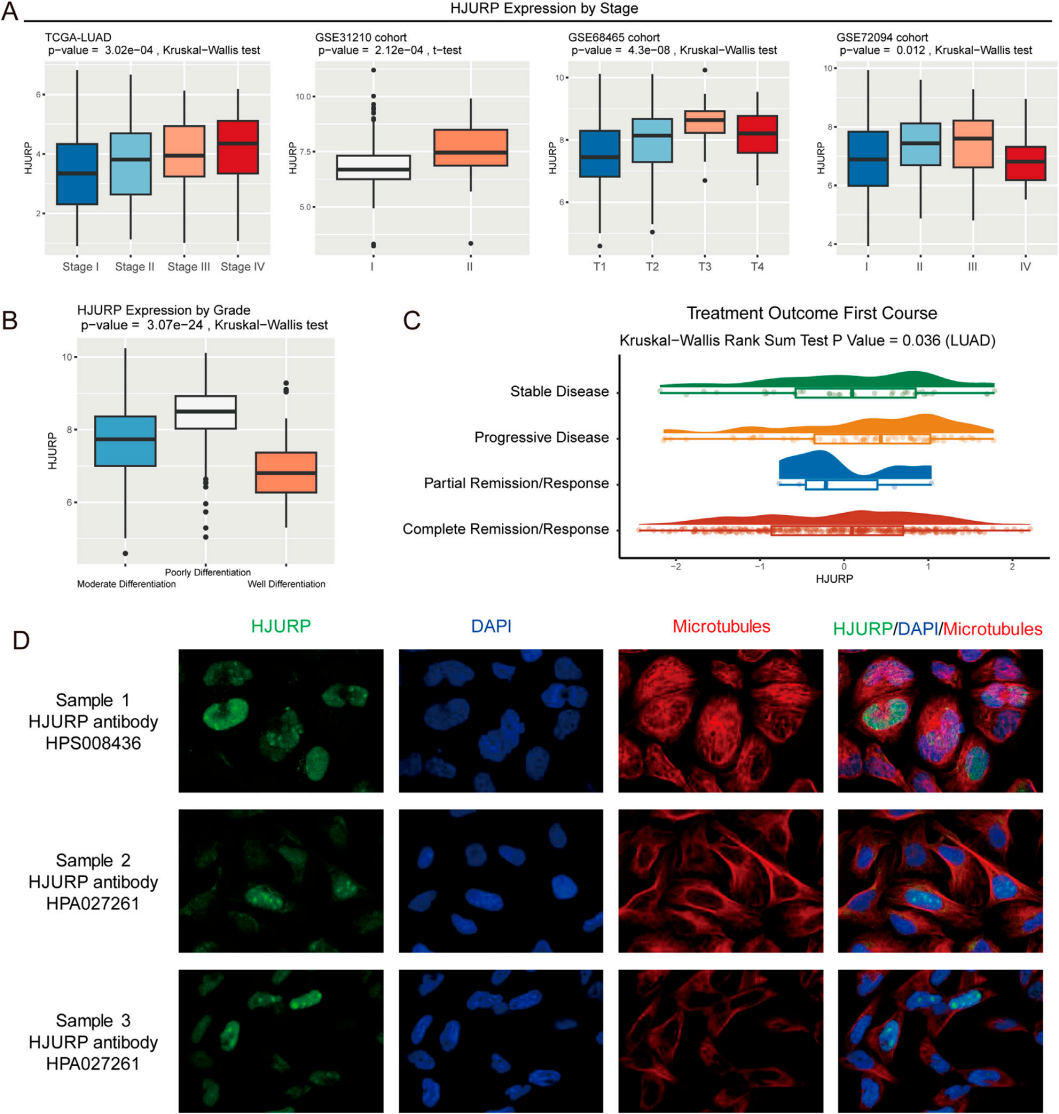
HJURP Expression Analysis by Tumor Stage, Differentiation, and Treatment Outcome. **(A)** HJURP expression comparing by tumor stage in TCGA-LUAD, GSE31210, GSE68465, and GSE72094 cohorts; **(B)** HJURP expression comparing by differentiation grade in TCGA-LUAD samples; **(C)** HJURP expression comparing by treatment outcome in the TCGA-LUAD cohort. **(D)** Immunofluorescent staining picture shows that HJURP mostly localized in the nucleoplasm and nucleoli.

HJURP expression increased with advanced tumor stage (TCGA-LUAD: *p* < 0.001, GSE31210: *p* < 0.001, GSE68654: *p* = 0.012, GSE72094: *p* = 0.012, Figure 4A). Compared to well-differentiated LUAD, poorly differentiated samples exhibited significantly higher HJURP expression (*p* < 0.001, Figure 4B). Additionally, we observed that patients with progressive disease in the TCGA-LUAD cohort had the highest HJURP expression in their tumor tissues (*p* = 0.036, Figure 4C). These results indicate that HJURP is closely associated with tumor progression, with males, smokers, and Asians being high-risk groups for HJURP-influenced LUAD development.

In further research, we conducted multivariate Cox regression analyses in different cohorts to eliminate confounding factors affecting HJURP’s prediction of LUAD prognosis (Table 1). In the TCGA-LUAD cohort, age over 70 years (*p* < 0.01), tumor stage (all other stages vs Stage I: *p* < 0.001), and HJURP expression (*p* < 0.001) were independent prognostic factors. In the GSE31210 cohort, age (*p* = 0.014), tumor stage (Stage II vs Stage I: *p* < 0.001), and HJURP expression (*p* = 0.042) were independent prognostic factors. In the GSE68465 cohort, age (*p* = 0.004), tumor grade (well vs moderate differentiation, *p* = 0.029), tumor T stage (T3 vs T1: *p* = 0.003, T4 vs T1: *p* = 0.004), tumor N stage (N1 vs N0: *p* < 0.001, N2 vs N0: *p* < 0.001), and HJURP expression (*p* < 0.001) were independent prognostic factors. In the GSE72094 cohort, gender (*p* = 0.003), tumor stage (Stage II vs Stage I: *p* = 0.004, Stage III vs Stage I: *p* < 0.001, Stage IV vs Stage I: *p* = 0.013), and HJURP expression (*p* < 0.001) were independent prognostic factors. Overall, multivariate regression analysis further confirmed HJURP expression as a predictor of LUAD prognosis, its high expression being a risk factor for poor prognosis.

**TABLE 1:** Multiple Cox regression analysis for LUAD patients.

TCGA-LUAD cohort	HR	95% CI	*p*_value
Gender			
Female	ref.		
Male	0.814	1.717–3.406	0.324
Age			
<70	ref.		
≥70	1.747	3.143–14.358	0.01*
Smoking			
Non-Smoker	ref.		
Smoker	0.668	1.379–4.003	0.279
Ever Smoker	1.239	1.924–10.428	0.511
Stage			
Stage I	ref.		
Stage II	3.523	8.322–349.787	1.20E-06*
Stage III	3.942	10.93–662.667	7.44E-08*
Stage IV	4.115	7.656–4100.615	8.18E-05*
HJURP	1.376	3.233–5.022	8.54E-05*

GSE31210 cohort	HR	95% CI	*p*_value
Age			
<70	ref.		
≥70	5.001	4.022–6.38E07	0.014*
Gender			
Female	ref.		
Male	1.014	1.472–14.264	0.977
Stage			
I	ref.		
II	3.686	6.139–1787.223	3.09E-04*
Smoking			
Never	ref.		
Ever	1.002	1.449–14.992	0.996
Genetic alteration			
ALK-fusion +	ref.		
EGFR mutation +	0.714	1.166–27.553	0.667
EGFR/KRAS/ALK -	1.227	1.292–358.084	0.798
KRAS mutation +	0.395	1.06–14.588	0.342
HJURP	1.385	2.749–6.655	0.042*

GSE68465 cohort	HR	95% CI	*p*_value
Age			
<70	ref.		
≥70	1.502	3.122–7.263	0.004*
Gender			
Female	ref.		
Male	1.283	2.639–5.449	0.08
Grade			
Moderate Differentiation	ref.		
Poorly Differentiation	0.986	2.071–3.801	0.927
Well Differentiation	1.656	2.862–13.553	0.029*
Race			
Asian	ref.		
Black or African American	3.027	1.817–4.64E06	0.181
Others	3.025	2.059–3.16E05	0.13
White	3.618	2.382–3.53E06	0.077
T stage			
T1	ref.		
T2	1.137	2.305–4.701	0.414
T3	2.177	3.706–37.25	0.003*
T4	2.788	3.988–275.287	0.004*
N stage			
N0	ref.		
N1	2.374	5.674–25.729	6.24E-08*
N2	3.806	13.953–243.476	9.97E-13*
Margin			
Negative	ref.		
Positive	0.995	1.547–9.706	0.991
HJURP	1.386	3.227–5.159	1.44E-04*

GSE72094 cohort	HR	95% CI	*p*_value
Age			
<70	ref.		
≥70	1.324	2.422–7.26	0.173
Gender			
Female	ref.		
Male	1.818	3.394–14.933	0.003*
Stage			
I	ref.		
II	2.066	3.547–29.127	0.004*
III	3.435	8.174–274.5	8.65E-07*
IV	2.832	3.489–612.267	0.013*
Race			
Black	ref.		
Other	0.966	1.17–387.269	0.97
White	1.011	1.367–26.303	0.985
Smoking			
1Never	ref.		
Ever	1.043	1.564–11.425	0.922
Missing	1.701	1.957–74.325	0.263
HJURP	1.332	3.098–4.807	6.04E-04*

### Distribution of HJURP in tumor tissues and cells

**FIGURE 5:**
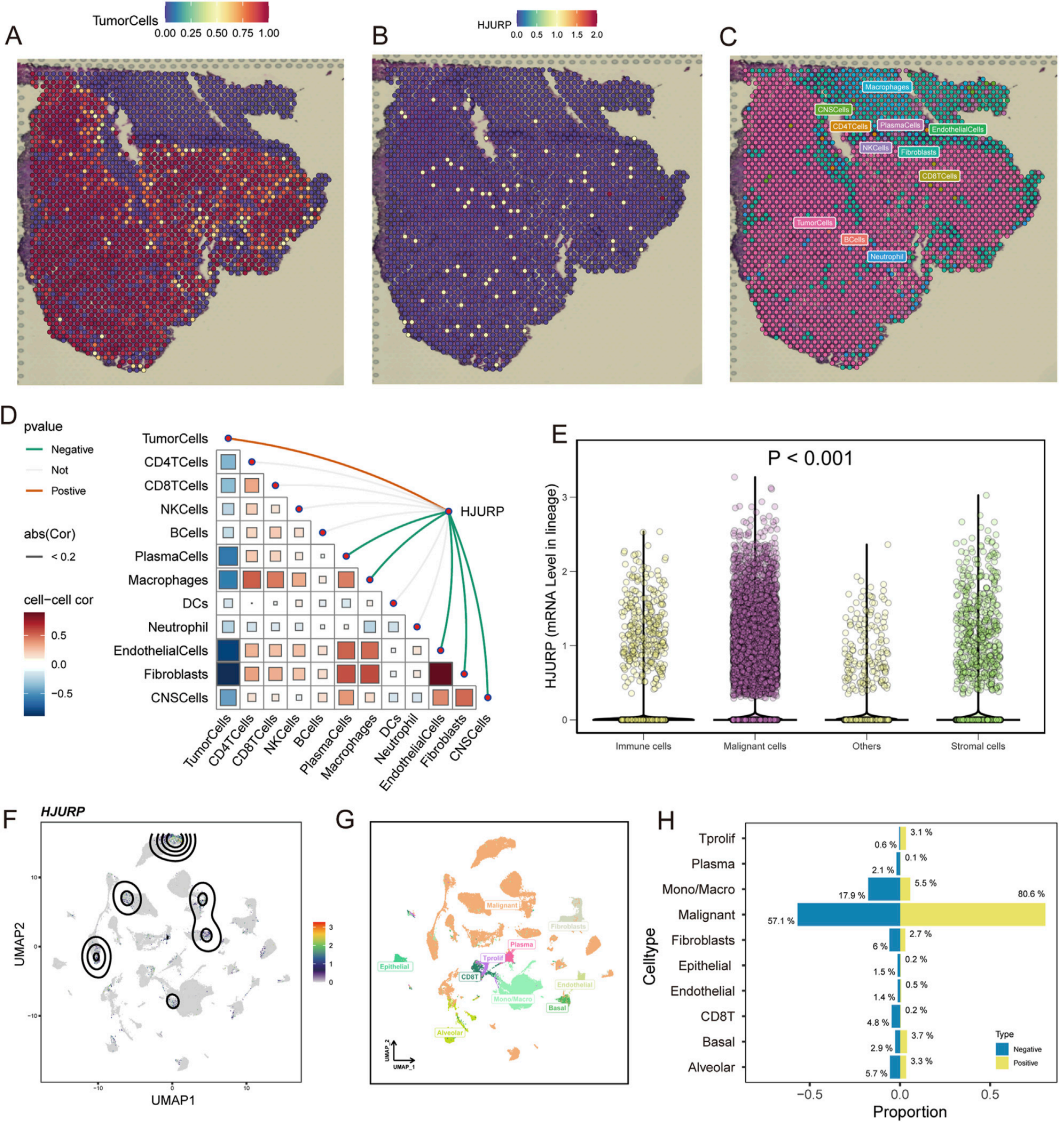
Spatial Transcriptomics and Single-Cell Sequencing Analysis of HJURP Expression in LUAD Tumor Tissues. **(A)** Spatial distribution of tumor cells in LUAD tissues; **(B)** Spatial distribution of HJURP expression in LUAD tissues; **(C)** Spatial distribution of immune cells in LUAD tissues; **(D)** Correlation analysis of HJURP expression with various cell types in LUAD tissues; **(E)** HJURP mRNA levels in different cell lineages in lung cancer tissues by single-cell sequencing data; **(F)** UMAP visualization of HJURP expression in single-cell sequencing data; **(G)** UMAP visualization of different cell types in single-cell sequencing data; **(H)** Proportion of cell types with positive HJURP expression in lung cancer tissues.

With the immunofluorescent staining picture provided by HPA, we observed that HJURP mostly localized in the nucleoplasm and nucleoli (Figure 4D), which can reflect its potential function in the regulation of genetic alteration. Using spatial transcriptomics data, we observed that in LUAD tumor tissues (Figure 5A), the regions of HJURP expression (Figure 5B) correspond to tumor areas rather than regions populated by immune cells (Figure 5C). This indicates that HJURP is predominantly expressed in tumor cells. Additionally, we found that HJURP expression levels positively correlate with tumor cell density and negatively correlate with plasma cells, macrophages, endothelial cells, and fibroblasts (Figure 5D). In single-cell sequencing data based on lung cancer tissues, HJURP was significantly more highly expressed in malignant cells compared to immune cells and stromal cells (*p* < 0.001, Figure 5E). Specifically, we observed that regions with high HJURP expression in single-cell sequencing data highly overlap with regions of malignant cells (Figures 5F,G). Among cells with positive HJURP expression, malignant cells accounted for 80.6% (Figure 5H).

### Potential functions of HJURP in LUAD development

**FIGURE 6:**
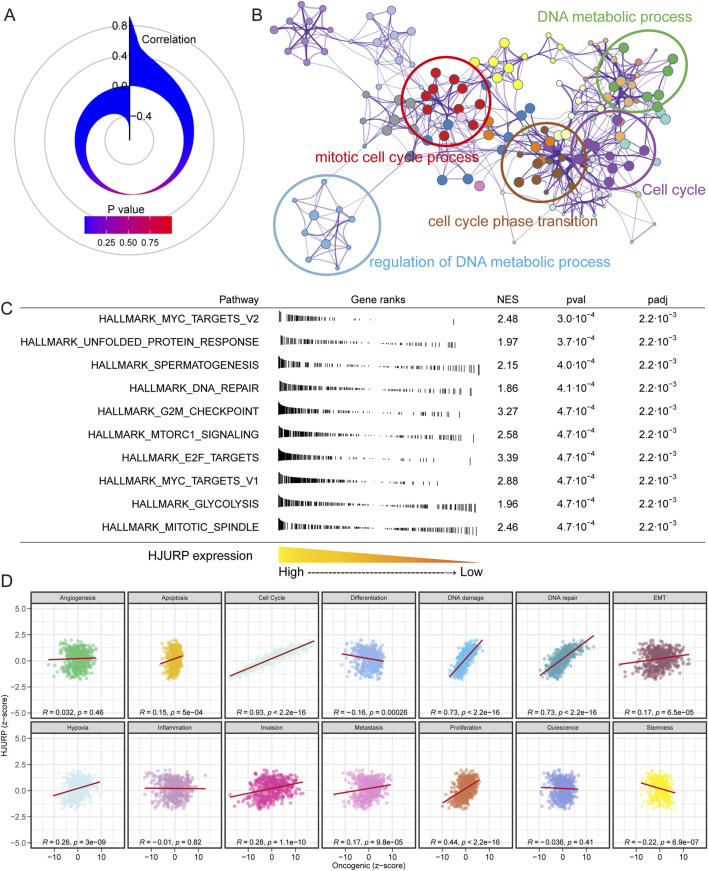
Correlation and Functional Enrichment Analysis of HJURP Expression in LUAD. **(A)** Correlation between HJURP expression and over 20,000 other genes; **(B)** Biological function enrichment analysis of the top 200 genes most correlated with HJURP expression; **(C)** Pathway activation analysis in patients with high versus low HJURP expression using the FGSEA algorithm; **(D)** Correlation of HJURP expression with the activation levels of 14 tumor development-related signaling pathways.

We calculated the expression correlation between HJURP and over 20,000 other genes (Figure 6A). We selected the top 200 genes with the highest expression correlation for biological function enrichment analysis. We found that HJURP may influence DNA metabolic processes, mitotic cell cycle processes, cell cycle phase transitions, and the cell cycle (Figure 6B). Using the FGSEA algorithm, we assessed the significantly different activation levels of signaling pathways between patients with high and low HJURP expression. In tumors of patients with high HJURP expression, cell cycle-related pathways were significantly activated, including MYC targets, G2M checkpoint, mitotic spindle, and E2F targets, as well as DNA repair, unfolded protein response, and glycolysis (Figure 6C). In further studies, we calculated the activation levels of 14 tumor development-related signaling pathways and evaluated their correlation with HJURP expression (Figure 6D). We found that HJURP was significantly positively correlated with the activation of cell cycle (R = 0.93, *p* < 0.001), DNA damage (R = 0.73, *p* < 0.001), DNA repair (R = 0.73, *p* < 0.001), cell proliferation (R = 0.44, *p* < 0.001), hypoxia (R = 0.26, *p* < 0.001), invasion (R = 0.28, *p* < 0.001), and metastasis (R = 0.17, *p* < 0.001) pathways, and showed a negative correlation with tumor differentiation (R = −0.16, *p* < 0.001) and stemness (R = −0.22, *p* < 0.001). In summary, HJURP may promote tumor progression in LUAD by influencing pathways related to the cell cycle and DNA damage repair.

### HJURP, genetic alteration and immunocytes infiltration

**FIGURE 7:**
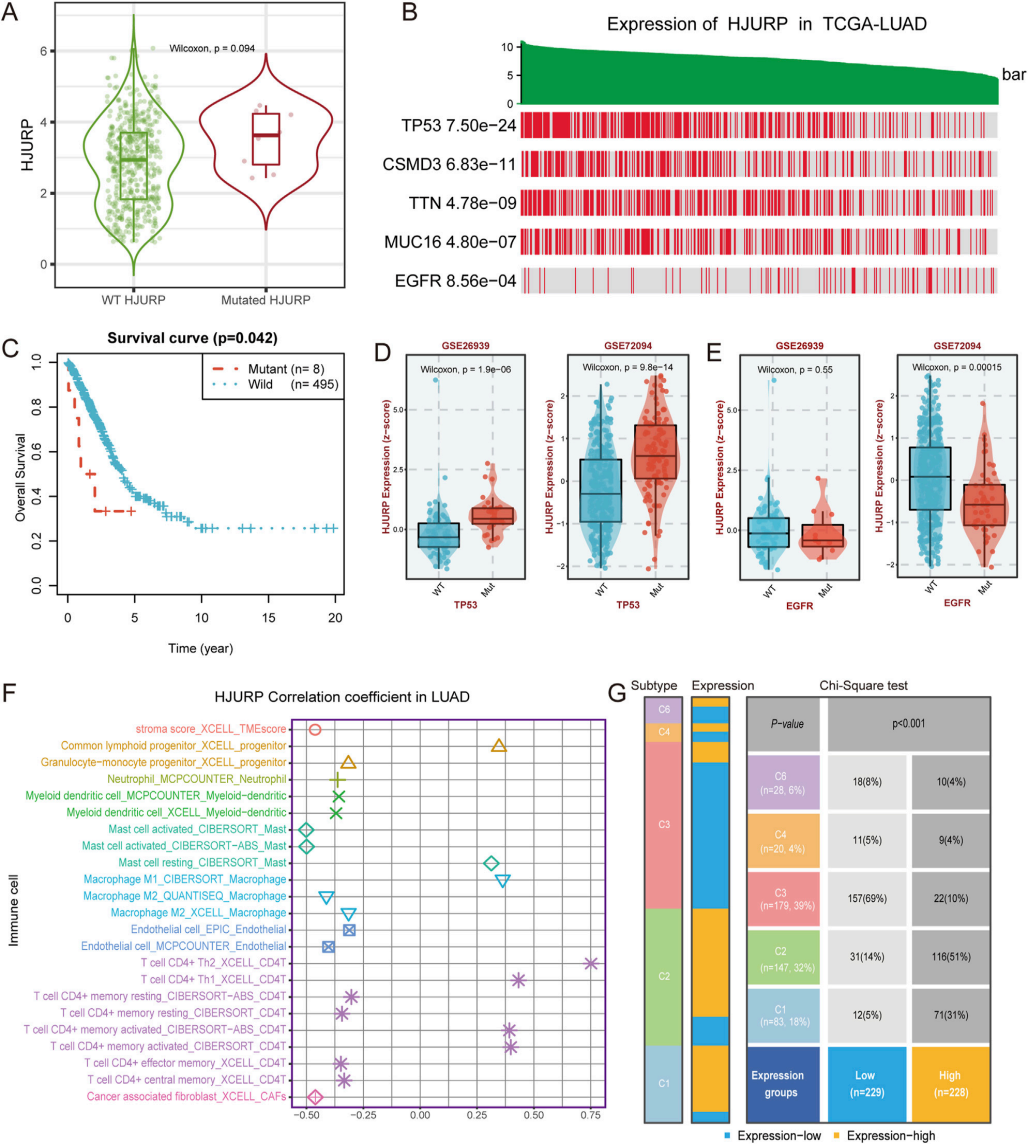
HJURP Expression, Gene Mutations, and Immune Cell Infiltration in LUAD **(A)** Expression of HJURP in wild-type and mutated HJURP samples; **(B)** HJURP expression in TCGA-LUAD samples with mutations in various genes; **(C)** Survival curve comparing overall survival between mutant and wild-type HJURP patients; **(D)** Validation of HJURP expression in TP53-mutated samples in GSE26339 and GSE72094 cohorts; **(E)** Validation of HJURP expression in EGFR-mutated samples in GSE72094 cohort; **(F)** Correlation of HJURP expression with immune cell infiltration in LUAD; **(G)** Distribution of HJURP expression groups in six immune subtypes of LUAD.

Tumor development and progression are closely related to gene mutations and immune cell infiltration. Mutation of HJURP gene can alter its expression, with mutated HJURP exhibiting higher expression levels (*p* = 0.094, Figure 7A). HJURP mutation are also associated with poorer prognosis (*p* = 0.042, Figure 7C), likely due to its increased expression levels. Additionally, we found that HJURP expression is closely correlated with a series of gene mutations. Patients with high HJURP expression showed higher mutation frequencies in TP53, CSMD3, TTN, and MUC16 genes, while the mutation frequency of the EGFR gene was lower (*p* < 0.001, Figure 7B). These findings were validated in external cohorts, where HJURP expression was significantly elevated in TP53-mutated samples (all *p* < 0.001, Figure 7D) and decreased in EGFR-mutated samples (*p* < 0.001 for GSE72094, *p* = 0.55 for GSE26939, Figure 7E).

Regarding the correlation between HJURP expression and immune cell infiltration, our analysis revealed that high HJURP expression was significantly positively correlated with the infiltration of CD4-positive T cells, such as Th2 and Th1 cells, as well as M1 macrophages, while it was negatively correlated with M2 macrophages (Figure 7F). These results suggest that high HJURP expression may be associated with a pro-inflammatory state in the tumor microenvironment. Further analysis of the distribution of high and low HJURP expression groups in six immune subtypes showed that LUAD patients with high HJURP expression were more likely to belong to the IFN-γ dominant (C2) subtype (Figure 7G), which exhibited a high proliferation rate that may override an evolving type I immune response (Thorsson et al., 2018). HJURP expression also showed a positive correlation with IFN-γ (R = 0.33, *p* < 0.001, Supplementary Figure S2).

### HJURP indicates potential precision therapy of LUAD

**FIGURE 8:**
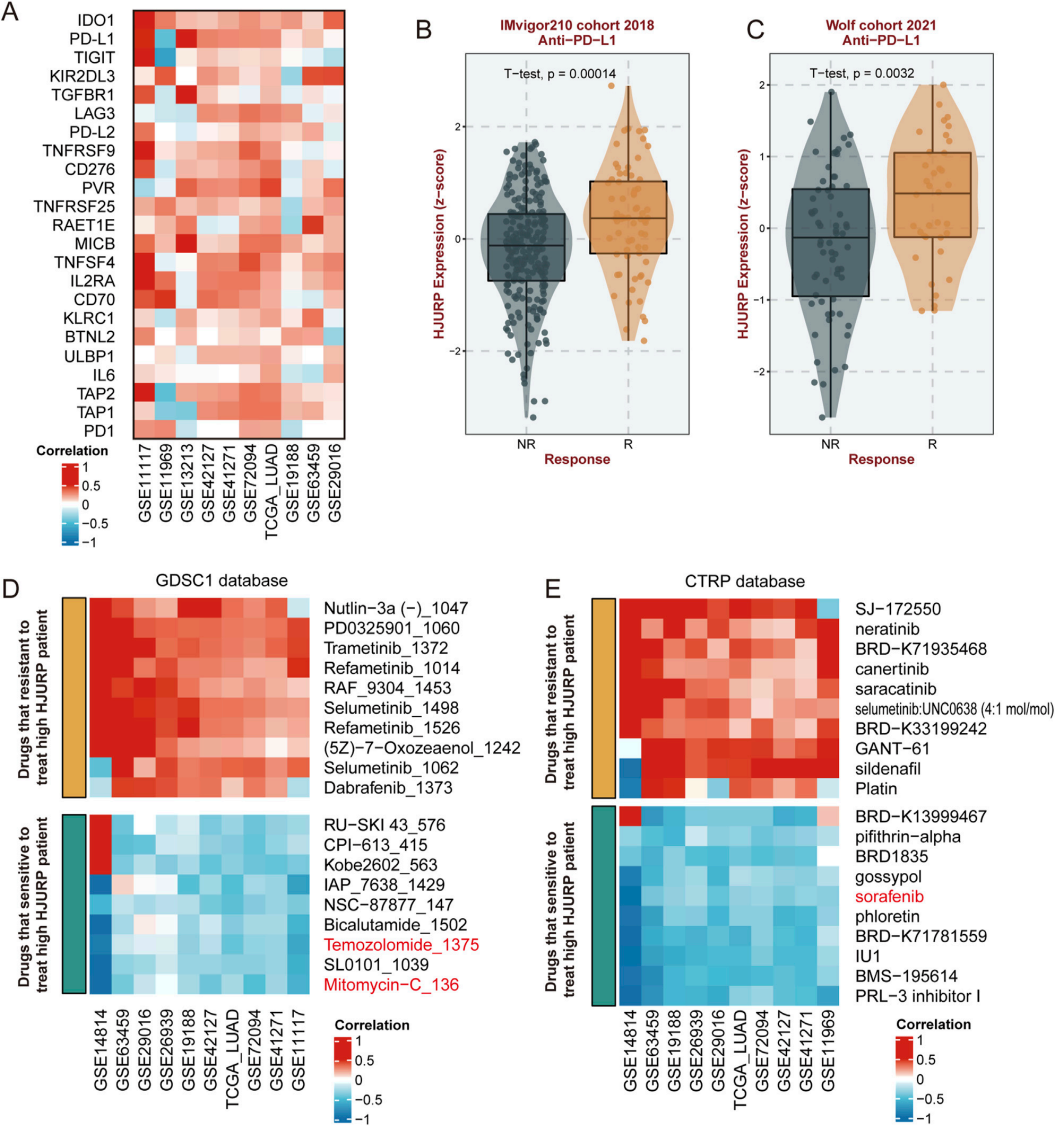
Correlation of HJURP Expression with Immune Checkpoints and Drug Sensitivity. **(A)** Correlation of HJURP expression with various immune checkpoint genes across multiple cohorts; **(B)** HJURP expression in responders (R) and non-responders (NR) to anti-PD-L1 treatment in the IMvigor210 cohort (2018); **(C)** HJURP expression in responders (R) and non-responders (NR) to anti-PD-L1 treatment in the Wolf cohort (2021); **(D)** Correlation of HJURP expression with drug sensitivity in the GDSC1 database; **(E)** Correlation of HJURP expression with drug sensitivity in the CTRP database.

Based on previous research findings, we observed that HJURP expression is positively correlated with the activation of cell proliferation, DNA damage repair pathways, and certain immune cell infiltration and immune response activation. Therefore, we further analyzed potential precision therapy strategies influenced by HJURP expression. We examined the correlation between a series of immune checkpoint genes and HJURP expression and found a significant positive correlation between HJURP expression and immune checkpoints such as PD-L1, PD-L2, IDO1, and MICB across multiple cohorts, while the correlation with PD1 expression was less pronounced (Figure 8A). Further analysis revealed that in patients responding to anti-PD-L1 treatment, HJURP expression was significantly higher compared to non-responders (IMvigor210 cohort 2018: *p* < 0.001, Figure 8B; Wolf cohort 2021: *p* = 0.0032; Figure 8C), which was not the case for anti-PD1 treatment (Supplementary Figure S3).

Regarding potential chemotherapy treatments, we identified from the GDSC1 database that commonly used drugs such as mitomycin, and temozolomide may be effective against tumors with high HJURP expression (Figure 8D). Additionally, from the CTRP database, we found that sorafenib could potentially have therapeutic effects (Figure 8E). These three common anti-tumor drugs already be applicated in the clinical treatment of LUAD, while others predicted agents should be further validated.

## Discussion

In this study, we identified Holliday Junction Recognition Protein (HJURP) as a significant prognostic biomarker for lung adenocarcinoma (LUAD). Using univariate Cox regression analysis across four independent cohorts (TCGA-LUAD, GSE31210, GSE68465, and GSE72094), we consistently found HJURP to be associated with poor overall survival (OS). Kaplan-Meier survival analysis reinforced these findings, showing significantly higher risks of OS in patients with elevated HJURP expression in all cohorts analyzed.

Our comprehensive analysis revealed that HJURP is not only associated with decreased survival rates but also closely linked to various clinical phenotypes. High HJURP expression was more prevalent in male patients, smokers, and Asians, indicating demographic-specific impacts. Additionally, elevated HJURP levels were associated with advanced tumor stages and poorly differentiated tumor samples, suggesting its role in tumor progression. The prognostic value of HJURP was widely reported in tumors. Yang et al. reported that HJURP was ectopically upregulated in Cholangiocarcinoma (CCA) compared with the para-tumor tissues, the high expression of HJURP was correlated with low overall survival rates of including intrahepatic CCA and perihilar CCA, but not in distal CCA(30). Kang et al. revealed that for patients with surgically resected colorectal cancer, patients with high expression of HJURP had significantly reduced cancer-specific survival rates compared to those with low HJURP expression (Kang et al., 2020). In breast cancer, HJURP expression levels are higher than in normal breast tissue. HJURP mRNA levels are significantly associated with estrogen receptor, progesterone receptor, Scarff-Bloom-Richardson grade, age, and the Ki67 proliferation index. Additionally, patients with higher HJURP levels exhibit increased sensitivity to radiotherapy (Hu et al., 2010). Chen et al. already discussed the prognostic value of HJURP in LUAD and its relationship with immune infiltration (Chen et al., 2022). In our study, we have employed a more comprehensive and innovative approach, our results not only corroborate the findings of Chen et al., but also provide new insights into the role of HJURP in LUAD. Specifically, we revealed that HJURP is one of the 19 prognostic genes from four clinical cohort from different study, and confirmed its prognostic value among OS, DSS, DFP and PFI. In addition, with the data of spatial transcriptomics and single-cell sequencing, we provide the new insight of the location of HJURP, that it’s most expressed in the tumor malignant cells, but less in immunocytes. Meanwhile, we also revealed that the mutation of HJURP linked with the elevated gene expression, and resulted in the poor prognosis. For the potential response to precis therapy, high level of HJURP might reflect the response to anti-PD-L1 therapy. These new findings add to the understanding of HJURP’s function in this context.

The molecular mechanisms by which HJURP influences LUAD progression appear multifaceted. HJURP’s involvement in DNA metabolic processes, cell cycle regulation, and mitotic processes were highlighted by our functional enrichment analyses. Specifically, pathways such as MYC targets, G2M checkpoint, and DNA repair were significantly activated in tumors with high HJURP expression. These findings align with previous reports that underscore the role of HJURP in maintaining genomic stability and promoting cell proliferation. The activation of HJURP appears to play a pivotal role in the immortality of cancer cells. HJURP is considered a potential downstream target of ataxia telangiectasia mutated signaling, and its expression is upregulated by DNA double-strand breaks (DSBs) (Kato et al., 2007). Furthermore, Serafim et al. demonstrate that HJURP is recruited to DSBs through a mechanism requiring chromatin PARylation and promotes epigenetic alterations that facilitate DNA repair. The incorporation of HJURP at DSBs promotes the turnover of H3K9me3 and HP1, thereby enhancing DNA damage signaling and DSB repair (Serafim et al., 2024). In prostate cancer, HJURP increased the ubiquitination of cyclin-dependent kinase inhibitor one via the GSK3β/JNK signaling pathway, decreasing its stability and thereby promoting cell proliferation (Lai et al., 2021). Chen et al. also reported that HJURP can promote hepatocellular carcinoma proliferation by destabilizing p21 via the MAPK/ERK1/2 and AKT/GSK3β signaling pathways (Chen et al., 2018).

Our study also explored the relationship between HJURP expression and genetic alterations in LUAD. High HJURP expression correlated with higher mutation frequencies in critical genes like TP53, CSMD3, TTN, and MUC16, while showing a lower frequency of EGFR mutations. These correlations suggest that HJURP might interact with specific genetic pathways to modulate LUAD pathogenesis. Overexpression of HJURP in senescent cells can partially overcome cellular senescence. Conversely, downregulation of HJURP in young cells leads to premature senescence, while knockdown of p53 can abolish the senescence phenotypes induced by the reduction of HJURP (Heo et al., 2013). TP53 mutations may lead to increased TP53 expression, suggesting that the HJURP gene and TP53 mutations might have a synergistic effect in LUAD development and progression.

In the spatial transcriptomic analysis, we observed that HJURP is predominantly expressed in tumor cells, with minimal expression in immune cells, including macrophages. Further analysis of bulk data revealed that HJURP expression does not significantly correlate with the activation of the Inflammation pathway. Specifically, our analysis indicated that HJURP expression is positively correlated with M1 macrophages, as well as Th1 and Th2 cells, and also positively correlated with M2 macrophages. However, the correlation between HJURP and the anti-inflammatory Th2 cells is stronger. In summary, HJURP in LUAD shows a generally low correlation with immune cell infiltration, with its expression most strongly associated with anti-inflammatory Th2 cells. Additionally, we reviewed relevant literature, which consistently highlights that high infiltration of M2 macrophages is associated with tumor progression and poor prognosis in LUAD (Zhang et al., 2011; Shikanai et al., 2023; Guo et al., 2019). Regarding PD-L1, the results show that HJURP is positively correlated with PD-L1 expression across most datasets, the upregulation of PD-L1 can protect tumors from immune cell attacks (Cui et al., 2024). Therefore, these patients are more suitable for anti-PD-L1 therapy and tend to achieve favorable outcomes. Given the substantial role of HJURP in LUAD, its potential as a therapeutic target is promising. Our analysis indicates that HJURP expression correlates positively with several immune checkpoint genes, including PD-L1, PD-L2, IDO1, and MICB, which are crucial for immune evasion. This relationship suggests that patients with high HJURP expression might benefit from immune checkpoint inhibitors, particularly anti-PD-L1 therapies. Additionally, our study identified several chemotherapeutic agents, such as mitomycin-C, temozolomide and sorafenib, of which that already be applicated in the clinical treatment of LUAD, indicating that could be effective against high HJURP-expressing tumors, paving the way for personalized treatment strategies. Mitomycin-C is an antitumor antibiotic that inhibits DNA synthesis by producing DNA cross-links which halt cell replication and eventually cause cell death (Bradner, 2001), temozolomide is a chemotherapy drug that works by alkylating DNA, which damages its structure and eventually kills the cell (Zhang et al., 2012), sorafenib blocks tumor proliferation and growth by inhibiting the RAF/MEK/extracellular signal-regulated kinase pathway (Hendrixson et al., 2024), these function is consistent with our findings that higher HJURP links with activation of DNA repair and cell cycle signaling.

Our study has several limitations that should be acknowledged. First, although we identified HJURP as a prognostic biomarker for LUAD across multiple independent cohorts, the heterogeneity across the cohorts may introduce variability due to differences in patient demographics, disease stages, and treatment histories, which may affect the generalizability of our findings. Additionally, selection biases could have influenced the data, as publicly available cohorts may not fully represent the broader LUAD population, potentially skewing results. Third, while we explored HJURP’s role in LUAD progression and its association with immune cell infiltration, our findings on immune modulation and therapeutic response are based on bioinformatic predictions and correlations. Experimental validation, particularly *in vitro* and *in vivo* studies, is necessary to establish causal relationships and confirm HJURP’s role in immune evasion and response to immunotherapies. Future research should aim to address these limitations to strengthen the clinical utility of HJURP as a biomarker and therapeutic target in LUAD.

## Conclusion

In summary, HJURP emerges as a pivotal biomarker and potential therapeutic target in LUAD. Its high expression is consistently associated with poor prognosis, advanced disease stages, and specific genetic and immune profiles. Future studies should focus on validating these findings in larger cohorts and exploring the therapeutic efficacy of targeting HJURP in LUAD.

## Data Availability

The original contributions presented in the study are included in the article/Supplementary Material, further inquiries can be directed to the corresponding author.
