# Supplementary figures and images for: Holliday junction recognition protein (HJURP) could reflect the clinical outcomes of lung adenocarcinoma patients, and impact the choice of precision therapy
Source: Front Genet. 2024 Nov 22;15:1475511. doi: 10.3389/fgene.2024.1475511 (PMC11621083; doi:10.3389/fgene.2024.1475511)

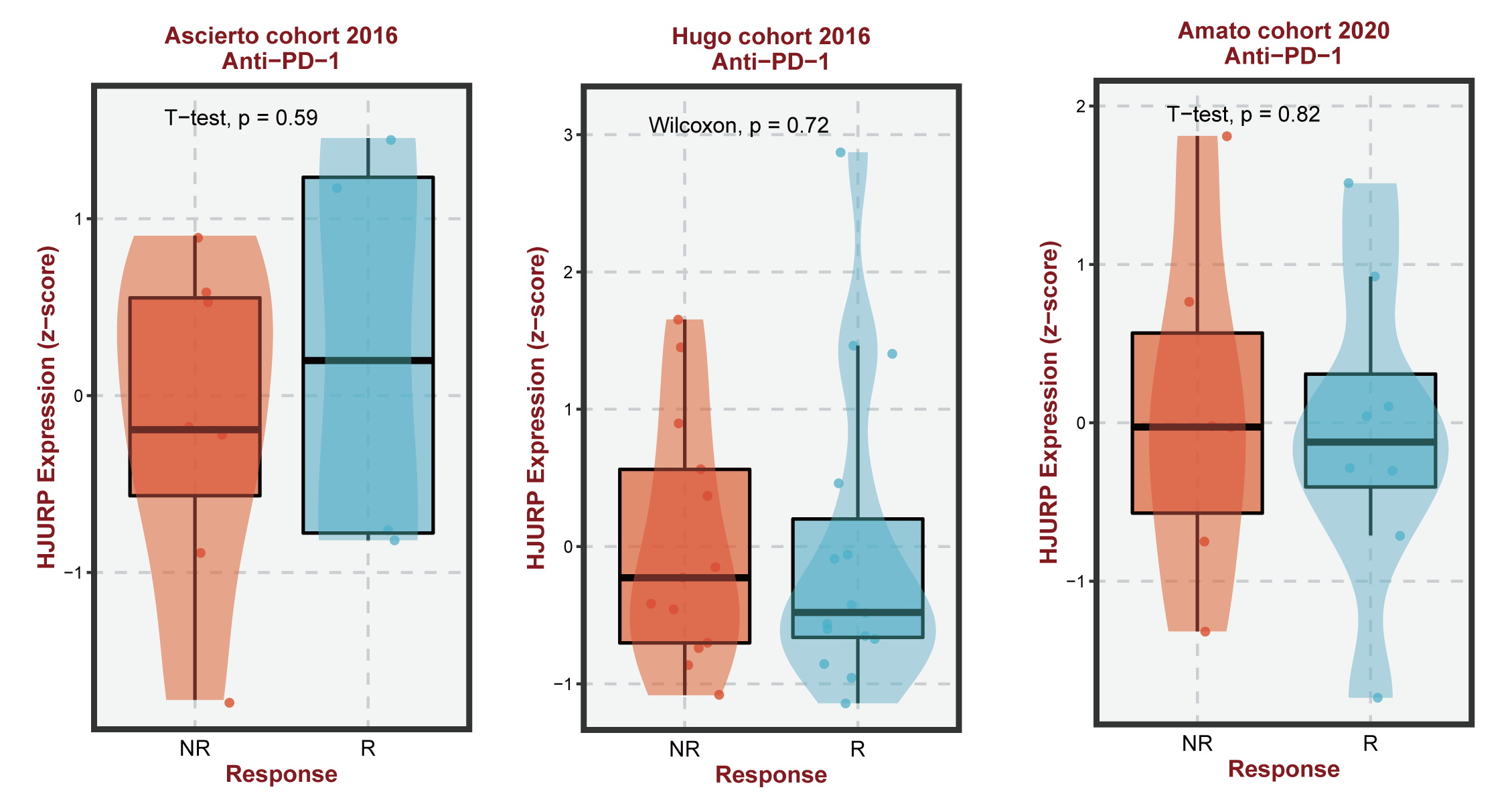

Supplement: Supplementary file 1 [file Image3.tif]

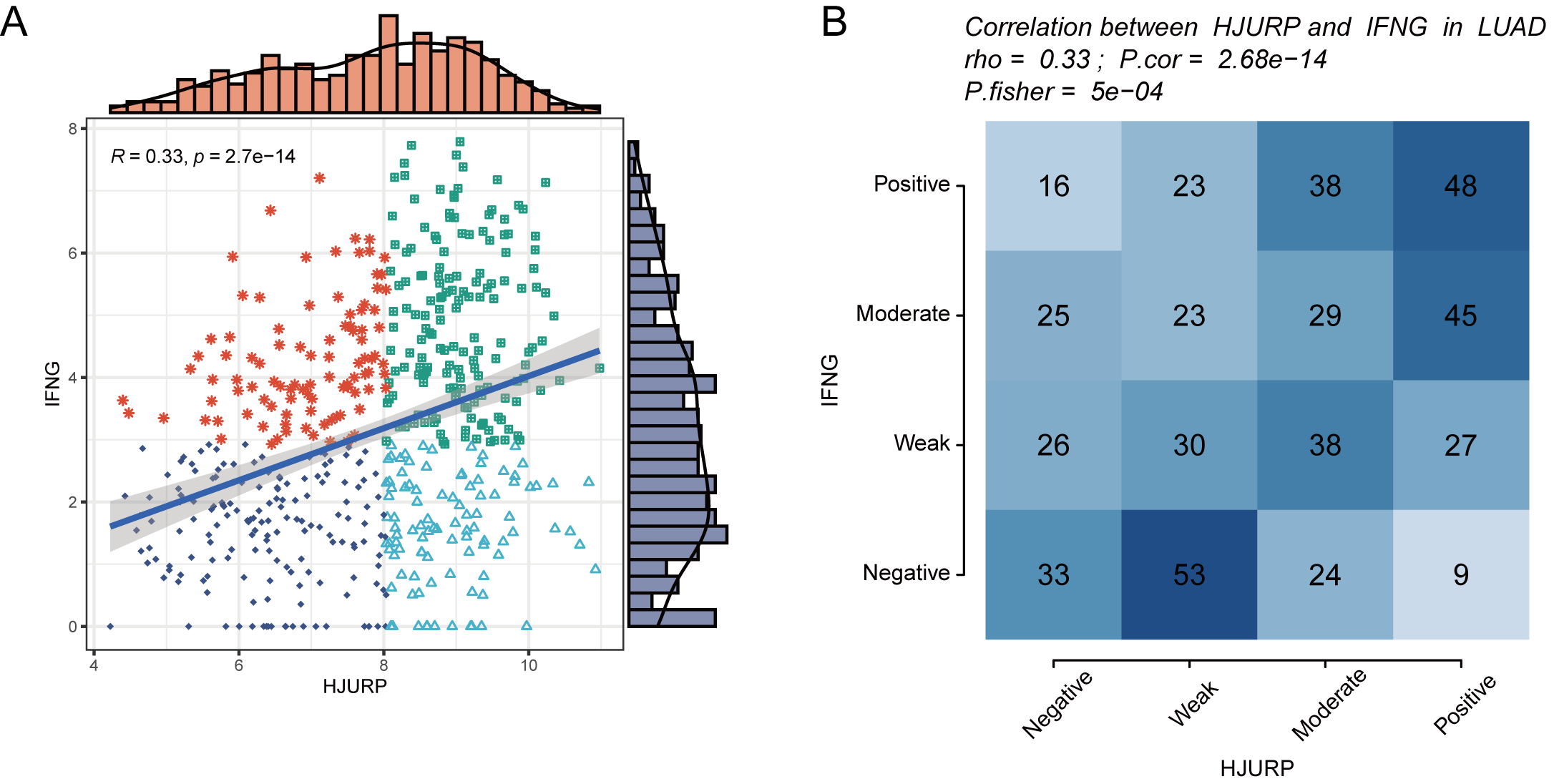

Supplement: Supplementary file 2 [file Image2.tif]

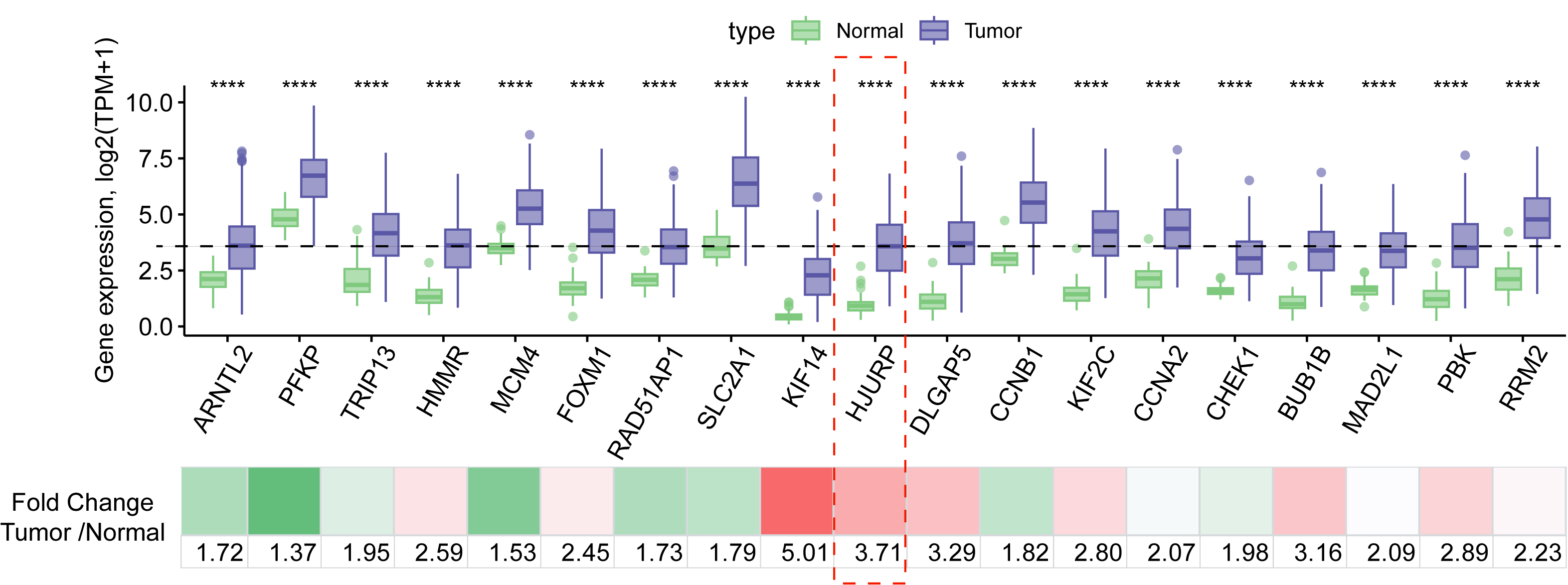

Supplement: Supplementary file 3 [file Image1.tif]
