# Supplementary material for: Holliday junction recognition protein (HJURP) could reflect the clinical outcomes of lung adenocarcinoma patients, and impact the choice of precision therapy
Source: Front Genet. 2024 Nov 22;15:1475511. doi: 10.3389/fgene.2024.1475511 (PMC11621083; doi:10.3389/fgene.2024.1475511)
Supplement: Supplementary file 4 [file Table2.docx]

**Supplementary Table S2.** Marker genes collected and curated for the annotation of immunocytes.

| **Immunocytes** | **Markers** |
| --- | --- |
| CD8T | BCL11B, CCL5, CD2, CD247, CD27, CD3D, CD3E, CD3G, CD6, CD69, CD7, CD8A, CD8B, CD96, CRTAM, CST7, CTSW, DPP4, DSC1, DUSP2, FAIM3, FLT3LG, GNLY, GPR171, GRAP2, GZMA, GZMB, GZMH, GZMK, GZMM, ICOS, IGKC, IL7R, ITK, KLRB1, KLRC3, KLRC4, KLRD1, KLRF1, KLRK1, LAG3, LCK, LEF1, LIME1, LTB, LY9, MAP4K1, MAP9, NCR3, NKG7, PIK3IP1, PRF1, PTGDR, PTPRCAP, PVRIG, RASA3, RPL3P7, SH2D1A, SIRPG, TCF7, TRAC, TRAT1, TRAV12-2, TRAV13-1, TRBC1, TRDC, UBASH3A, ZAP70 |
| Mono/Macro | AIF1, APOBEC3A, AQP9, ASGR1, ASGR2, BST1, C5AR1, CCR2, CD1D, CD33, CD68, CDA, CFP, CHST15, CLEC4A, CLEC7A, CREB5, CSF3R, FAM198B, FCN1, FES, FOSB, FPR1, FZD2, HCK, HK3, HNMT, HPSE, IGSF6, LILRA2, LILRA3, LILRB2, LST1, MEFV, MNDA, MS4A6A, NCF2, NFE2, NLRP3, NOD2, P2RY13, PADI4, RNASE2, RNASE6, S100A12, SLC15A3, TLR2, TLR7, TLR8, UPK3A, VNN1, VNN2, ACP5, ADAMDEC1, BHLHE41, CCDC102B, CCL18, CCL22, CCL7, CHI3L1, COL8A2, CSF1, CXCL3, CXCL5, CYP27A1, DCSTAMP, GPC4, MARCO, MMP9, PLA2G7, PPBP, QPCT, SLAMF8, SLC12A8, TNFSF14, TREM2, ACHE, APOL3, APOL6, ARRB1, CCL19, CCL5, CCL8, CCR7, CD38, CD40, CLIC2, CXCL10, CXCL11, CXCL13, CXCL9, CYP27B1, DHX58, EBI3, GGT5, HESX1, IDO1, IFI44L, IL2RA, KIAA0754, KYNU, LAG3, LAMP3, PLA1A, PTGIR, RASSF4, RSAD2, SIGLEC1, SLAMF1, SLC2A6, SOCS1, TNFAIP6, TNIP3, TRPM4, ALOX15, CCL13, CCL14, CCL23, CD209, CD4, CLEC10A, CRYBB1, FRMD4A, GSTT1, HRH1, HTR2B, NME8, NPL, PDCD1LG2, RENBP, WNT5B |
| Plasma | ABCB9, AMPD1, ANGPT4, ATXN8OS, C11orf80, CCR10, CD27, CD38, CD79A, DENND5B, EAF2, FCRL2, GNG7, GPR25, GUSBP11, HIST1H2AE, HIST1H2BG, HLA-DOB, IGHD, IGHE, IGHM, IGKC, IGLL3P, KCNA3, KCNG2, LIME1, LOC100130100, MAN1A1, MANEA, MAST1, MROH7, MZB1, P2RX5, PAX7, PDK1, PNOC, RASGRP3, REN, RGS13, RPL3P7, SIK1, SPAG4, ST6GALNAC4, TGM5, TMEM156, TNFRSF17, UGT2B17, ZBP1, ZNF165 |
| Fibroblasts | FAP, PDPN, MMP2, PDGFRA, THY1, MMP11, PDGFRL, TGFB3, COL1A2, DCN, COL3A1, COL6A1 |
| Endothelial | PECAM1, VWF, ENG |
| Tprolif | BCL11B, CD2, CD247, CD27, CD28, CD3D, CD3E, CD3G, CD6, CD7, CD8A, CD8B, CD96, CXCR6, FLT3LG, FYN, GIMAP4, GPR171, GZMK, GZMM, ICOS, ITK, LCK, LIME1, PRKCH, PSTPIP1, SH2D1A, SIRPG, TNFRSF9, TRAC, TRAT1, TRBC1, TRBC2, UBASH3A, ZAP70, AURKA, BIRC5, BUB1, CCNA2, CCNB1, CDC20, CDK1, CDKN3, FEN1, HMGB2, MCM2, MCM5, MCM6, MYBL2, NUSAP1, PCNA, PLK1, TOP2A, ZWINT |
